# Much Ado about Five Cents

**Published:** 1884-12

**Authors:** 


### MUCH ADO ABOUT FIVE CENTS.

TWO gentlemen were talking the other day of the great amount of detail work in the United States Treasury Department. As an instance of the scrupulous exactness with which the work in the Redemption Bureau is done, one of the gentlemen told the following story:

"Several years ago," he said, "I was living in the country, and had among my possessions an old desk. One day, in going through this I found four pieces of scrip money of small denominations which preceded the return of silver. Two of these pieces were quarters and a third was a ten-cent scrip. Each of these was in fair condition. The forth was a ragged and soiled bit of paper, with nothing but its size and feeling to indicate that it had ever been money. After studying it a long time, I concluded it was originally a five-cent scrip. I determined to send the three good pieces to the Treasury Department for redemption, more as an experiment than anything else. But my wife insisted that the ragged piece must go along with the rest. To humor her I enclosed it with the others, and a note asking that the scrip be redeemed

"A week or ten days later I was informed that there was a registered letter at the Post Office for me. I found a huge envelope with three red seals and the card of the Treasury Department upon it. In the envelope were several papers, three or four, with printed and written matter upon them. The papers were blanks which had been filled out and extracts from the law concerning the redemption of fractional currency. From the documents I learned that the three pieces I had supposed were redeemable were counterfeit and of no value, and that the only piece upon which I could base a claim against the Department was he tattered and torn five-cent scrip. Down in one corner of the big envelope was a smaller one, and in one corner of that was a bright nickel five-cent piece, which the United States Government had sent to me in return for my five-cent scrip."

**A Specific for Hiccough.**—Dr. Henry Tucker recommends, in the *Southern Medical Record*, the use of the following very simple remedy in the treatment of hiccough, namely: moisten granulated sugar with good vinegar. Of this give to an infant from a few grains to a teaspoonful. The effect, he says, is almost instantaneous, and the dose seldom needs to be repeated. He has used it for all ages—from infants of a few months old to those on the downhill side of life, and has never known it to fail. The remedy is certainly a very simple one, and although no theory is advanced to account for its wonderful action, it merits trial.

